# Earthing effects on mitochondrial function: ATP production and ROS generation

**DOI:** 10.1002/2211-5463.70062

**Published:** 2025-06-13

**Authors:** Cecilia Giulivi, Richard Kotz

**Affiliations:** ^1^ Department of Molecular Biosciences, School of Veterinary Medicine University of California Davis CA 95616 USA; ^2^ University of California Medical Investigations of Neurodevelopmental Disorders (M.I.N.D.) Institute Sacramento CA USA; ^3^ North American Science Associates (NAMSA) St. Louis Park MN USA

**Keywords:** ATP production, earthing, grounding, mitochondria, oxidative stress

## Abstract

Mitochondria are central to cellular energy production and the regulation of oxidative stress. Traditional methods for assessing mitochondrial ATP and reactive oxygen species (ROS) rely on metal probes, which unintentionally ground the system, confounding results. To investigate the impact of grounding on mitochondrial function, we utilized fluorescence‐based experiments to assess these mitochondrial outcomes under three conditions: wired (grounded), sham, and naïve. Mitochondria under grounded conditions produced significantly more ATP (by 5–11%), reduced ROS production (by 22–33%), and decreased mitochondrial membrane potential (by 5–6%) than sham and naïve. These findings indicate that grounding improves mitochondrial bioenergetics by reducing oxidative stress. Future research should explore the broader implications of grounding over time on mitochondrial health and its potential therapeutic applications.

## Graphical abstract

In contrast to sham and naive controls, grounded mitochondria not only exhibit significantly enhanced energy production but also demonstrate a remarkable reduction in membrane potential and oxidative stress. This suggests a profound improvement in mitochondrial health, presenting a promising avenue for therapeutic interventions.

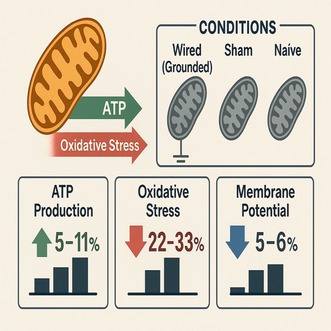

**Abbreviations**
ADP: adenosine diphosphate
ANCOVA: analysis of covariance (one‐way ANCOVA)
ANOVA: analysis of variance (one‐way ANOVA)
ARRIVE guidelines: Animal Research: Reporting of *In Vivo* Experiments guidelines
ATP: adenosine triphosphate
DAMPs: damage‐associated molecular patterns
ETC: electron transport chain
FCCP: carbonyl cyanide‐*p*‐trifluoromethoxyphenylhydrazone
HRP: horseradish peroxidase
MSHE buffer: mannitol, sucrose, EGTA, HEPES buffer
OCR: oxygen consumption rate
p‐HPA: *p*‐hydroxyphenylacetic acid
RCR: respiratory control ratio
ROS: reactive oxygen species
Tukey's HSD: Tukey's honestly significant difference (*post hoc* test)

Mitochondria are increasingly recognized as essential centers for various critical cellular processes, including energy metabolism [1, 2, 3], immune response [4, 5, 6], and signal transduction [7, 8, 9, 10]. These organelles are responsible for producing adenosine triphosphate (ATP), the primary energy currency of cells, and they also regulate the production of reactive oxygen species (ROS), which are significant contributors to oxidative stress [3, 11, 12, 13]. Dysfunction in these processes is associated with the development of numerous chronic and degenerative diseases, which genetic, environmental, and lifestyle factors can influence [11, 12, 13, 14].

Mitochondrial ATP production occurs through an oxidative phosphorylation process, which primarily relies on the electron transport chain (ETC). This process is essential for generating the energy needed for various cellular functions. However, during oxidative phosphorylation, ROS are also produced as a byproduct [15, 16]. While low levels of ROS can be beneficial and play critical roles in cellular signaling pathways, excessive or uncontrolled ROS levels can lead to significant cellular damage and oxidative stress [3, 11, 12, 13, 14, 17, 18, 19]. Imbalances in ROS production are increasingly recognized as important factors in the development of several health conditions. For example, they are implicated in aging, contributing to age‐related decline in cellular function [1, 20, 21, 22, 23, 24, 25, 26]. Additionally, elevated ROS levels are linked to a range of metabolic disorders, neurodegenerative diseases such as Parkinson's and Alzheimer's, and systemic diseases like diabetes and certain types of cancer [27].

In addition to their role in energy production and signaling, mitochondria also have crucial functions in the immune system [4, 5, 28, 29, 30, 31, 32]. Under conditions of cellular dysfunction, mitochondria can release damage‐associated molecular patterns (DAMPs), which are molecules that alert the immune system to potential threats [33]. This release of DAMPs can trigger inflammatory responses, thereby playing a significant role in the body's innate immunity [33, 34, 35, 36, 37, 38, 39].

These intricate interactions underscore the vital importance of assessing and understanding mitochondrial function, both for preventing diseases and developing effective treatment strategies. By targeting mitochondrial dysfunction and regulating ROS levels, it may be possible to influence the progression of various diseases and improve overall health outcomes.

Understanding how mitochondria produce ATP and ROS under varying conditions is crucial. Mitochondria generate ATP through oxidative phosphorylation while simultaneously producing ROS as byproducts. These reactive molecules can cause oxidative stress unless tightly regulated, contributing to aging and disease. Grounding (or earthing)—direct contact with the Earth's surface—is thought to impact mitochondrial function by influencing electron flow, potentially affecting both ATP synthesis and ROS levels.

Interestingly, many experimental procedures, such as those using polarography to measure oxygen consumption through a Clark‐type oxygen electrode [40, 41] and first applied to study mitochondrial oxygen uptake and oxidative phosphorylation by Chance and Williams [42], are performed under grounded conditions, stabilizing electrical noise and improving signal detection. However, little attention has been paid to how grounding might alter mitochondrial bioenergetics. Our study compared ATP and ROS production and coupling between electron transfer and ATP production in grounded versus non‐grounded mitochondria to explore whether grounding alters mitochondrial function.

Grounding, or earthing, refers to the direct contact of human or animal skin with the Earth's surface. This may occur through bare feet, hands, or grounding devices facilitating conductive interaction with the Earth's surface. Contact with the Earth neutralizes excessive oxidative stress and ROS, thereby influencing the body's physiology.

It is noteworthy that humans and all other animals have been in direct contact with the ground continuously until contemporary times, particularly in the 1950s when insulating synthetic‐soled footwear was introduced [43].

Earthing offers numerous wellness benefits—as evidenced by research based on clinical trials and randomized clinical trials—indicating that grounding individuals promptly improves their mood and decreases fatigue [44, 45], enhances vagal tone and heart rate variability [44], improves blood circulation and viscosity [44, 46], regulates cortisol levels [47], diminishes blood glucose [48], promotes better sleep [44, 47, 48], mitigates inflammation [49], and alleviates delayed‐onset muscle soreness or pain in general [44, 47, 49], among various other health benefits (https://earthinginstitute.net/research).

The observations above, including those regarding the effects of earthing on reducing muscle damage [50] without improving the energy cost of running or the physiological responses of elite athletes [51], prompted our investigation into the impact of ROS and ATP during grounding on mitochondria.

Our findings indicated that under grounded conditions, mitochondria produced less ROS than non‐grounded mitochondria, suggesting that grounding may help prevent oxidative damage to mitochondria over time, thereby minimizing oxidative stress. These results underscore the importance of grounding on mitochondrial outcomes and highlight the necessity of evaluating mitochondrial function under conditions that are grounded and not grounded to understand their natural bioenergetic state fully. This comparison may offer new insights into the potential therapeutic benefits or risks of grounding in the context of mitochondrial health and disease prevention.

## Materials and methods

### Animals

Male C57BL/6 (B6) mice (Jackson Laboratories, Sacramento, CA, USA) were kept in pathogen‐free conditions, according to the guidelines of the University of California, Davis Institutional Animal Care and Use Committee (IACUC). The University of California, Davis Institutional Review Board for the Protection of Animal Welfare (#21780) approved all experimental procedures and the ethics of this study. The study was carried out in compliance with the IACUC requirements and the ARRIVE guidelines [52]. Mice consumed *ad libitum* chow and water. To isolate mitochondria from livers, mice were humanely euthanized by CO_2_ inhalation.

### Mitochondria isolation and bioenergetics evaluation

All reagents were of analytical grade or higher. Mitochondria were isolated from the livers of 3‐month‐old male mice (strain C57BL/6). Mouse liver mitochondria were isolated as previously described [53, 54, 55]. Briefly, samples were homogenized in isolation buffer (0.22 m mannitol, 0.07 m sucrose, 1 mm EGTA, 10 mm HEPES, pH 7.4) with a handheld PRO Scientific Bio‐Gen PRO200 homogenizer at a 1:10 ratio (1 g wet weight tissue in 10 mL buffer). Subsequently, differential centrifugation was used to obtain mitochondria‐enriched fractions. Purified mitochondria from mitochondria‐enriched fractions were collected by differential centrifugation followed by Percoll gradient and multiple washes in MSHE buffer (220 mm d‐Mannitol, 70 mm sucrose, 0.5 mm EGTA, 2 mm HEPES, 0.1% fatty‐acid free bovine serum albumin, pH 7.4) followed by a final resuspension in 0.5 mL of 150 mm KCl (final protein concentration of 70 mg·mL^−1^). Protein was determined using the Pierce™ BCA protein assay kit and following the manufacturer's instructions. Oxygen consumption was evaluated using a Hansatech Clark‐type oxygen electrode (Hansatech, King's Lynn, UK) [54, 55, 56]. Mitochondria (150–300 μg protein) were added to the oxygen chamber in a buffer containing 0.22 m sucrose, 50 mm KCl, 1 mm EDTA, 10 mm KH_2_PO_4_, and 10 mm HEPES, pH 7.4. ATP‐driven oxygen consumption rates were evaluated in the presence of (i) 1 mm malate‐10 mm glutamate followed by 1 mm ADP, and then the addition of 5 μm rotenone and (ii) 10 mm succinate followed by the addition of 1 mm ADP, and then followed by the addition of 3.6 μm antimycin A. The activities of mitochondrial NADH‐linked ATP production and FADH_2_ linked ATP production were evaluated as the difference in oxygen uptake recorded before and after the addition of rotenone and antimycin A, respectively.

### Grounding setup

**Fig. 1:**
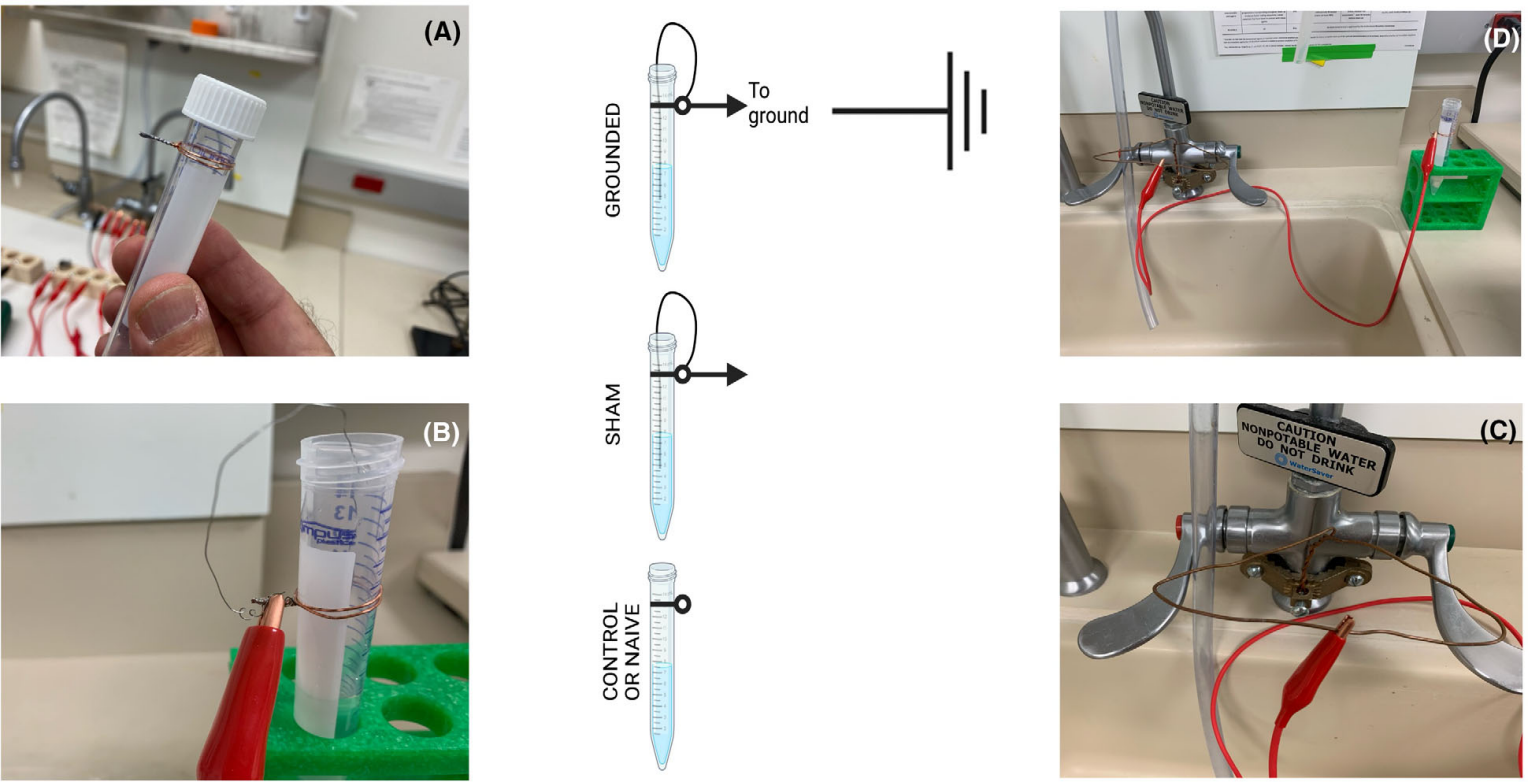
Experimental setup for grounding experiments. Counterclockwise: (A) Copper wires were coiled around conical 15 mL test tubes. (B) One end of a thin stainless steel wire was connected to the bundle of copper wire, while the other end formed a loop that was submerged in the solution inside the tube. Care was taken to ensure the thin wire did not touch the tube walls. A separate red cable with metal alligator clips at each end connected the copper wire (B) to a grounded faucet (C, D), which was verified using a multimeter. The center of the figure illustrates a schematic representation of the three experimental setups: grounded (top), sham (middle), and naïve (bottom). The arrow symbolizes the red cable with metal alligator clips.

Nine ml of MSHE buffer (0.21 m mannitol, 0.07 m sucrose, 1 mm EDTA, 1 mm EGTA, 10 mm HEPES, pH 7.4) was aliquoted in 9 × 15 mL conical plastic tubes, 6 of which were connected to a 14 cm long stainless steel 3 mm wire (submerged halfway into the 1 mL of buffer). Of the 9 conical tubes, 3 were grounded (stainless steel wire + cable connected to the sink faucet), 3 were sham (stainless steel wire + cable not connected to the faucet), and 3 were not grounded or control (no stainless steel wire, no cable connected to ground; Fig. 1). The ground condition was verified by using a handheld multimeter. A digital multimeter (Commercial Electric MS83010A manual‐ranging digital multimeter), set to continuity test mode with an audible output, was used to assess the integrity of each circuit. This test ensured that there were no breaks or unintended resistance in the electrical pathways. The audible output provided real‐time confirmation of a continuous connection, allowing efficient troubleshooting. Additionally, this method was used to verify that each tube was properly grounded, ensuring a secure and low‐resistance connection to the ground, which is critical for both safety and proper circuit functionality.

Throughout the text, mitochondrial states are mentioned. For clarity, in mitochondrial respiration, State 3, State 3u, and State 4 represent different mitochondrial activity phases observed during respirometry experiments. State 3 (also known as active respiration) reflects the maximal rate of mitochondrial oxygen consumption when ADP is available for phosphorylation. In this state, mitochondria are actively converting oxygen into ATP through oxidative phosphorylation, which represents their energy production capacity. State 3u (uncoupled State 3) occurs when uncouplers, such as FCCP, are introduced, dissipating the proton gradient across the inner mitochondrial membrane. This causes mitochondria to consume oxygen at a high rate without synthesizing ATP as the electron transport chain continues to operate, but ATP production is uncoupled from respiration. Lastly, State 4 (resting respiration) is characterized by lower oxygen consumption after the ATP produced in State 3 has been used. In this state, there is no ADP available, and respiration is limited to the basal oxygen consumption needed to maintain the mitochondrial membrane potential and perform basic cellular functions, without active ATP production. These states are crucial for assessing mitochondrial function, including the respiratory control ratio (State 3/State 4), which helps gauge the efficiency of mitochondrial coupling and overall bioenergetic health.

The incubation buffers were as follows for the three conditions: (i) non‐phosphorylating mitochondria (or idle): MSHE +10 mm succinate (for testing endogenous synthesis of ATP with endogenous ADP); (ii) phosphorylating mitochondria: MSHE +10 mm succinate +1 mm ADP (to test phosphorylating activity by providing substrate and ADP); and (iii) uncoupled mitochondria (i.e., uncoupling of electron transport from ATP synthesis): MSHE +10 mm succinate +3.6 μm Antimycin A + 20 nm FCCP. At *t* = 0 min, mitochondria were added to each tube at a final concentration of 0.3 mg·mL^−1^, thoroughly mixed, aliquots were taken at time zero (estimated at <5 min), and then incubated for 15 and 30 min at 22 °C. At these 3 time points, aliquots of 200 μL (60 μg protein) and 5 μL (1.5 μg protein) were transferred to, respectively, 1.8 mL of ROS assay buffer (0.1 m K_2_HPO_4_, 5 units·mL^−1^ horseradish peroxidase and 400 μm *p*‐hydroxyphenylacetic acid) and 45 μL of assay buffer (BioVision, proprietary) maintained on ice. Mitochondrial H_2_O_2_ production was determined fluorimetrically, using a previously described method [57]. Briefly, the reaction between horseradish peroxidase (HRP), *p*‐hydroxyphenylacetic acid (p‐HPA), and hydrogen peroxide (H_2_O_2_) follows a peroxidase‐catalyzed oxidation process that leads to the formation of a fluorescent product. HRP utilizes H_2_O_2_ as an oxidizing agent, forming an activated HRP‐peroxide complex capable of transferring electrons to suitable substrates. In this case, p‐HPA undergoes one‐electron oxidation by the HRP‐peroxide complex, forming oxidized p‐HPA, which subsequently dimerizes or polymerizes into a highly fluorescent product. The oxidized p‐HPA product exhibits strong fluorescence, which can be measured spectrophotometrically (typically with excitation around 320–350 nm and emission around 400–450 nm). The reaction proceeds via a radical‐based mechanism and is commonly used for detecting H_2_O_2_ in biological and enzymatic assays. The fluorescence intensity of the oxidized p‐HPA product correlates with the amount of H_2_O_2_ present, allowing for sensitive detection [58]. At the end of the experiment, for the ROS measurement [59], 200 μL sample—in triplicates—from the 2 mL reaction tube were loaded in a well of a 96‐well plate, and fluorescence was recorded (excitation and emission wavelengths at 315 and 410 nm, respectively) after incubation for 30 min at 37 °C. ROS production was calculated based on a calibration curve obtained with a stock solution of 100 μm H_2_O_2_ at 0–3 μm concentrations. Negative (MSHE buffer only) and positive (0.5 μm H_2_O_2_) controls were also loaded onto the microplate in triplicates.

At the end of the experiment, ATP levels were evaluated spectrophotometrically (570 nm) with a BioVision kit (cat # K354) following the manufacturer's instructions after a 30‐min incubation at 25 °C. A calibration curve of ATP ranging from 0–10 nmol·well^−1^ was used for ATP quantification. Negative (MSHE buffer only) and positive (5 nmol ATP/well) controls were run in triplicates.

The mitochondrial transmembrane potential (Δψ) was assayed fluorometrically (507 and 529 nm, excitation and emission wavelengths) using (0.4 μm) Rhodamine 123 (Fisher Scientific) as a probe in an Infinite M200 (Tecan, Morrisville, NC). This fluorescent dye is used to assess Δψ in isolated mitochondria. Its cationic and lipophilic nature allows it to accumulate within mitochondria in response to their membrane potential selectively [60]. The fluorescence was evaluated in aliquots at 0 and 30 min (technical triplicates and two biological replicates). As a positive control for ΔΨ = 0, 3 μm FCCP was added at the end of each measurement to collapse ΔΨ by eliminating the proton motive force across the mitochondrial inner membrane. The potential was calculated using the Nernst equation, considering the partition of dye in the mitochondria vs. in the media devoid of mitochondria. The dye Rhodamine 123 is used as a probe for mitochondrial membrane potential because it is well characterized, causes no loss of mitochondrial coupling, and is not toxic at low concentrations [61, 62].

### Statistical analysis

All experiments were run in two biological replicates and three technical triplicates. The one‐way ANOVA (analysis of variance) test was employed to determine whether there were statistically significant differences among three or more experimental conditions. In this case, the conditions included phosphorylating, non‐phosphorylating, uncoupled or grounded, sham, and naïve groups. The test assessed whether the mean values of a dependent variable differed across these groups. Following the ANOVA, Tukey's *post hoc* analysis was conducted to identify specific group differences. Tukey's test controls for the family‐wise error rate and is particularly useful when performing multiple comparisons, ensuring that significant differences between group means are not due to random variation. Additionally, linear regression analysis was performed to explore relationships between variables, where Pearson's correlation coefficient was used to quantify the strength and direction of the linear relationship between two continuous variables. All statistical analyses were executed using JMP software version 17.0, a widely used statistical tool for data visualization and analysis.

## Results

### Quality control of mitochondrial preparation

The quality control of mouse liver mitochondrial preparations was evaluated before performing any experiments to assess the impact of grounding on mitochondrial ATP and ROS production. All mitochondrial samples had respiratory control ratios (RCR) 12 ± 3 using standard polarography equipment. The samples were used within 2 hours after preparation, and there was no significant decline in the RCR <9 during this period. Under these conditions, the rates of oxygen uptake with malate‐glutamate and succinate under phosphorylating conditions were 26 ± 3, 39 ± 4 nmol, and 27 ± 3 nmol of O_2_ consumed × (min × mg mitochondrial protein)^−1^. The rate of oxygen consumption under non‐phosphorylating conditions (succinate only; State 4) was 3.5 ± 0.5 nmol O_2_ × (min × mg mitochondrial protein)^−1^. The rate of ROS production in State 4 was 0.60 ± 0.02 nmol H_2_O_2_ × (min × mg protein)^−1^. If the rate under State 4 reflects the proton leak and electron leak from the mitochondrial inner membrane, then the electron leak (exit of electrons throughout the electron transport chain before the reduction of oxygen to water at cytochrome *c* oxidase resulting in superoxide anion production) represents a minor fraction of the total oxygen consumption rate in State 4 (6‐to‐1 ratio), in agreement with others [63].

### Validation of experimental setup and approaches

All experiments were performed at 22 °C to avoid unspecific activation of proteases and lipases, which could compromise mitochondrial integrity throughout the experiment (~30–60 min). Higher temperatures may require larger amounts of protease, kinase, phosphatase, and lipase inhibitors to prevent degradation, with unexpected and sometimes detrimental impacts on mitochondrial bioenergetics. Additionally, while overall oxygen uptake rates are higher at 37 °C, the slower oxygen consumption rate at 22 °C ensures that mitochondria (or cells) do not experience hypoxia or anoxia during the evaluation period. This was critical as the grounding setup did not provide a real‐time reading of oxygen levels in the test tubes. Temperature has a well‐known impact on mitochondrial function, including ATP‐linked oxygen consumption rates (OCR). As shown by others, Arrhenius plots of OCR in State 3 with succinate vs. 1/T support the temperature dependence of mitochondrial activity (Fig. 1A). These results indicated a proportional decrease in OCR at lower temperatures as the electron transport chain components slowed down [64]. In addition, the activation energy calculated from that plot (14.7 ± 0.4 cal·mol^−1^) was within published values for the 17–30 °C temperature range (15.4 to 16 cal·mol^−1^) [64], validating our results. Furthermore, the 20–25 °C range has been widely used in mitochondrial studies, including Chance's seminal work in the 1950s‐60s, where experiments ranged from 4 to 25 °C, with most conducted at 25 °C [65, 66, 67, 68, 69, 70]. Thus, the selection of performing bioenergetic assessments at 22 °C preserves mitochondrial integrity, obtains reliable respiration measurements while avoiding unwanted, excessive enzymatic degradation, and prevents hypoxic conditions.

We designed experiments that utilized spectroscopic techniques (fluorescence) to assess ROS and ATP, as most mitochondrial studies employ metal probes to measure these parameters, which can alter the mitochondrial solution and thus confound the experiments. Both ATP synthesis and H_2_O_2_ production were initially measured under baseline conditions to establish reference values. This step was crucial to ensure that any subsequent changes observed in ATP and ROS levels could be accurately attributed to grounding rather than variations in the mitochondrial preparations. Detailed protocols for quantifying ATP and H_2_O_2_ were followed to ensure precision and reliability in the measurements. Analysis of the variance showed a main effect of mitochondrial state on ATP production [*F*(2, 23) = 1062.44, *P* < 0.0001, η^2^ = 0.989]. As expected, ATP production was the highest in phosphorylating mitochondria when a substrate (succinate) and ADP were provided at non‐limiting concentrations (State 3; mean ± SD = 401 ± 35 nmol × (min × mg mitochondrial protein)^−1^ compared to States 4 or 3u). Post hoc analyses using Tukey's HSD indicated that ATP production in State 3 was higher than that in State 4 (*P* < 0.0001) and State 3u (*P* < 0.0001; Fig. 1B). Conversely, under conditions of endogenous (limiting) ADP (but with excess succinate or State 4) or uncoupled conditions with antimycin (State 3u with antimycin), ATP production was negligible [19 ± 3 and 18 ± 2 nmol × (min × mg mitochondrial protein)^−1^, respectively] and statistically not different from each other (*P* = 0.996).

In terms of ROS production, analysis of the variance showed a main effect of mitochondrial states on the H_2_O_2_ production rate [*F*(2,24) = 29.47, *P* < 0.0001, η^2^ = 0.711]. The mitochondrial ROS production was maximal under antimycin‐supplemented uncoupled conditions (State 3u with antimycin = 0.18 ± 0.02 nmol H_2_O_2_ × (min × mg protein)^−1^ compared to phosphorylating (0.10 ± 0.02) and non‐phosphorylating (0.13 ± 0.02) conditions (*P* < 0.0001 and *P* = 0.0002, respectively; Fig. 1C)). The rate of ROS production under phosphorylating conditions was also different and lower than that obtained under non‐phosphorylating conditions (*P* = 0.035; Fig. 1C).

Considering a current P/O = 1.636 for succinate (1.636 molecules of ATP are produced for each oxygen atom reduced to water during the oxidation of succinate) [71, 72, 73], the fraction of oxygen utilized for ROS production was 2.2% under non‐phosphorylating conditions and 0.08% under phosphorylating conditions. Consistent with these values and according to current literature, a very small fraction of oxygen consumed by mitochondria is used for ROS production, typically estimated to be between 0.15% and 2% of total oxygen consumption, with most studies placing it closer to the lower end of this range at around 0.1–0.2% [74] for mitochondria in State 3.

The values of these outcomes indicated that not only the mitochondria preparations that would be used for the grounded wiring settings passed the quality control for intactness and coupling but also the experimental setups and approaches.

### Grounding experiments

**Fig. 2:**
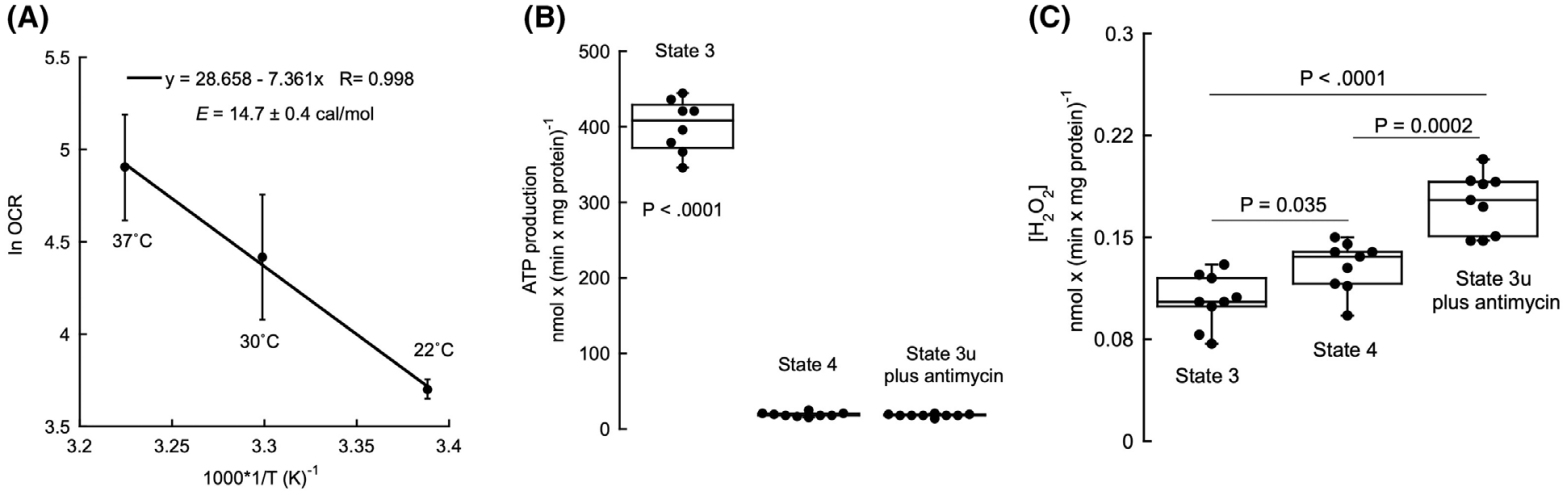
Arrhenius plot for State 3 with succinate, ATP, and hydrogen peroxide production by isolated, purified mouse liver mitochondria. (A) The oxygen consumption rate (OCR) with succinate and ADP (State 3) by isolated mouse liver mitochondria was evaluated at three temperatures. The Arrhenius plot was constructed using the natural log of the mean rates of OCR at each temperature tested. The data were fitted to a linear model (equation shown on the panel; Pearson's correlation *r*(2) = −0.998; *P* = 0.036). Error bars represent the standard deviation. For Panels B, C, isolated mitochondria were incubated with succinate and ADP (phosphorylating; State 3), with the substrate (succinate) only (non‐phosphorylating; State 4), and with the FCCP uncoupler (carbonyl cyanide‐*p*‐trifluoromethoxyphenylhydrazone) and antimycin (uncoupled; State 3u with antimycin). ATP (Panel B) and hydrogen peroxide production (Panel C) were evaluated in aliquots (taken in triplicates) at 0, 15, and 30 min, and the rates were calculated as the slopes of a linear regression fitting. Statistical analyses were performed with ANOVA (analysis of variance) followed by Tukey's post hoc. The box plots in panels B and C are designed to enclose 50% of the data within each box, with the median value (defined as the data value located halfway between the smallest and largest values) represented as a line. The top and bottom of the box indicate the limits of ±25% of the variable population. The lines extending from the top and bottom of each box depict the minimum and maximum values within the dataset that fall within an acceptable range. Any value outside this range, referred to as an outlier, is shown as an individual point (an outlier is defined as any point whose value is either greater than the upper quartile +1.5 * interquartile distance or less than the lower quartile − 1.5 * interquartile distance). Outliers are included in the calculations for the box plot. All experiments were run in at least two biological replicates and three technical triplicates.

For grounding experiments, copper wires were coiled around the necks of conical 15‐mL test tubes. A thin stainless steel wire connected to the copper coil had a loop submerged in the solution within the tube, ensuring it did not touch the tube walls. Grounding was established by connecting the copper wire to a faucet. The system was fully linked to the faucet in the grounding condition, confirmed by a multimeter as properly grounded (see Methods). The sham condition replicated this setup but did not have a grounding connection, acting as a control for potential non‐grounding effects. The naïve condition included no external wiring or grounding, serving as a baseline for comparison (Fig. 2).

**Fig. 3:**
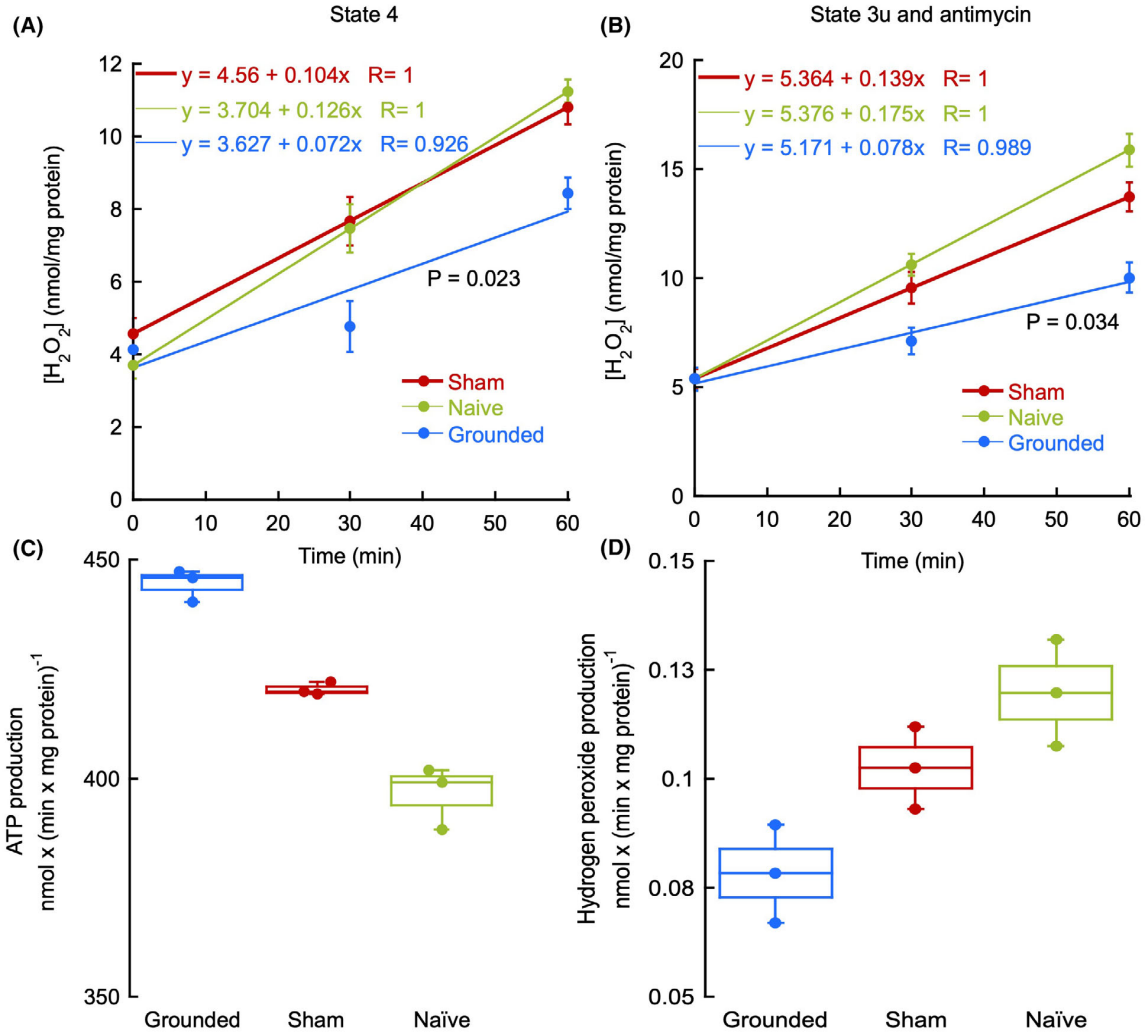
Effect of grounding on ATP and hydrogen peroxide production and respiratory control ratio in phosphorylating mitochondria. Hydrogen peroxide production was evaluated at the indicated time points in mitochondria incubated with succinate (A) and succinate, FCCP, and antimycin (B). Aliquots were taken in triplicates under each condition: grounded, sham, and naïve. Linear regression equations are shown in each panel. The P‐value corresponds to a one‐way ANCOVA (analysis of covariance). The rates of ATP production (C) and hydrogen peroxide production (D) were evaluated in phosphorylating mitochondria (State 3). *P* values were obtained from ANOVA analysis followed by Tukey's *post hoc*. All experiments were run in two biological replicates and three technical triplicates. For panels A and B, error bars represent the standard deviations. The box plots in panels C and D show 50% of the data within each box, with the median indicated by a line. The box limits represent ±25% of the variable population. Lines extending from the boxes depict the minimum and maximum values within an acceptable range. Values outside this range, called outliers, are shown as individual points (defined as any point greater than the upper quartile +1.5 * interquartile distance or less than the lower quartile − 1.5 * interquartile distance). Outliers are included in box plot calculations.

First, we assessed the time course for H_2_O_2_ production by mouse liver mitochondria under State 4 and 3u with antimycin to evaluate the linear response within the time window (Fig. 3A and Fig. 3B). Under the three conditions, the responses under State 4 were linear with correlation coefficients >0.93 [Pearson's correlation *r*(2) >0.93, *P* < 0.001]. Similar results were obtained with State 3u and antimycin. One‐way analysis of covariance (ANCOVA) indicated that the H_2_O_2_ production under grounded settings was significantly different from and lower than those observed under sham and naïve conditions for State 4 (*P* = 0.023) and State 3u with antimycin (*P* = 0.034).

The rates of ATP production (Fig. 3C) and H_2_O_2_ production (Fig. 3D) were evaluated in mouse liver mitochondria under phosphorylating conditions (State 3) and under the three settings (grounded, sham, and naïve). [Correction added on 24 July 2025 after first online publication: This sentence has been corrected.] Significant differences were observed under grounded conditions compared to sham and naïve. Analysis of the variance showed a main effect of grounding on ATP production [*F* (2, 6) = 78.598, *P* < 0.0001, *P*η^2^ = 0.963]. Post hoc analyses using Tukey's HSD indicated that the ATP production was 444 ± 2 nmol ATP × (min × mg protein)^−1^ under grounded conditions, significantly higher than that under sham (420 ± 1; *P* = 0.002) and naïve conditions (396 ± 7; *P* < 0.0001; Fig. 3C). Aligned with the ATP production results, analysis of the variance showed a main effect of grounding on H_2_O_2_ production rates [*F*(2,6) = 10.803, *P* = 0.010, pη^2^ = 0.783]. Post hoc analyses using Tukey's HSD indicated that the ROS production was not different between sham and naïve conditions [in nmol H_2_O_2_ × (min × mg protein)^−1^ 0.103 ± 0.009 and 0.12 ± 0.01; *P* = 0.213], but both were higher than that under grounded conditions (0.08 ± 0.01; *P* = 0.079 vs. sham; *P* = 0.009 vs. naïve; Fig. 3D).

In sum, the ATP production was the highest under grounded conditions, accompanied by the lowest mitochondrial ROS production and the highest coupling of ATP production to oxygen uptake or electron flow through the electron transport chain.

Considering that grounding resulted in a statistically significant increase in ATP production compared to sham and naïve conditions (5 to 11%), but with a more substantial impact on ROS production (decreased by 22 to 33%) than in sham and naïve conditions, and that research studies indicated that even a modest decrease in membrane potential (<13%) can lead to a 1.2‐ to 2‐fold increase in respiration rate and substantial (by 80%) reduction in H_2_O_2_ production while exerting minimal effects on State 3 respiration [75, 76], we evaluated the mitochondrial membrane potential under the three conditions. The ΔΨ under naïve or sham conditions was not different (−174 ± 9 and − 175 ± 10 mV), whereas under grounded conditions, it was significantly lower (−165 ± 8 mV). All three ΔΨ dropped to −20 ± 6 mV values in the presence of FCCP.

## Discussion

The findings from our study highlight the significant impact of grounding on mitochondrial function, particularly in ROS generation, which consequently affects the coupling of electron transfer to ATP production. Using fluorescence‐based assays to evaluate these parameters, we successfully avoided the confounding effects that traditional metal probes introduced by grounding the mitochondrial solution. This methodological approach enabled us to isolate and accurately assess the influence of grounding on mitochondria. Currently, no experimental work has unequivocally demonstrated that grounding alters mitochondrial bioenergetic parameters. While various claims regarding the health benefits of grounding exist, including its potential effects on oxidative stress, these assertions have largely remained speculative due to a lack of direct empirical support. Our study aimed to address this knowledge gap by providing experimental data to evaluate whether grounding has measurable effects on mitochondrial respiration, membrane potential, and oxidative stress. Rather than assuming a causal relationship, we systematically tested whether grounding exerts any bioenergetic influence under controlled conditions. By doing so, we aimed to establish a foundation for future investigations into the potential physiological relevance of grounding at the mitochondrial level.

Our study demonstrated that grounding led to a slight decrease in mitochondrial membrane potential, which accounts for the reduction in ROS production and the increase in ATP production. Although the decrease was ~5–6% and could be considered a minor biological impact, it is necessary to remember that the proton leak rates in mammalian mitochondria increase exponentially with ΔΨ and that the increase in proton conductance across the mitochondrial membrane is also non‐ohmic with ΔΨ increases. Proton and electron leak are intricately linked, as superoxide production is highly sensitive to the decrease in Δproton due to proton leak [77]. Our results aligned with those of others, indicating that even modest decreases in membrane potential can lead to a substantial decrease in H_2_O_2_ production [75, 76]. This aligns with our findings and supports the hypothesis that electrical grounding may modulate mitochondrial bioenergetics via subtle changes in membrane potential.

Mitochondrial respiration and membrane potential are often measured in respiratory chambers fitted with electrodes, such as the commonly used oxygraph systems. These systems rely on oxygen consumption rates as a proxy for mitochondrial activity. Still, they do not always account for potential artifacts introduced by mitochondrial grounding—a phenomenon where mitochondria physically interact with surfaces, potentially altering their bioenergetic behavior. When mitochondria are in suspension, as in many high‐resolution respirometry experiments, they can maintain their native electrochemical gradients. However, when mitochondria come into contact with chamber surfaces, such as the electrodes in an oxygraph, this grounding can influence charge distribution, impacting membrane potential measurements and potentially altering oxygen consumption rates. In systems where mitochondria are deposited onto surfaces or enclosed in microchambers, grounding effects might lead to membrane potential dissipation, causing artificial depolarization or altered proton leak dynamics. This could result in underestimated respiratory control ratios or misinterpretation of mitochondrial coupling efficiency. Understanding these possible effects is critical for ensuring that measured respiration and potential accurately reflect physiological mitochondrial behavior. Comparing electrode‐based O_2_ consumption measurements with alternative approaches, such as fluorometric potential‐sensitive dyes or isolated vesicle systems, may help clarify how mitochondrial grounding influences bioenergetic assessments.

Reduced levels of ROS are advantageous since excessive ROS can cause oxidative stress, damaging cellular components such as lipids, proteins, and DNA. These observations suggest that grounding positively influences mitochondrial bioenergetics by reducing oxidative stress through decreased ROS production. Thus, grounding may offer an exciting therapeutic target for altering the pathophysiology associated with mitochondrial superoxide production and diseases characterized by increased oxidative stress resulting from dysfunctional mitochondria.

Our results support the hypothesis that grounding provides therapeutic potential across various contexts. Decreased levels of ROS reduce oxidative damage, which may slow processes associated with aging and chronic diseases, which are associated with a decline in mitochondrial function.

In conclusion, these findings underscore the significance of grounding in mitochondrial research and suggest potential therapeutic benefits of grounding for managing conditions related to mitochondrial dysfunction and oxidative stress. Future studies should investigate the long‐term effects of grounding and its possible applications in preventive and clinical contexts.

## Conflict of interest

All authors have disclosed any financial or other interests related to the submitted work that could impact the authors' objectivity or influence the article's content. No financial or nonfinancial competing interests could compromise this publication's objectivity, integrity, or value by affecting the authors' judgment and actions in data presentation, analysis, and interpretation. C.G. serves as an Editorial Board Member of Scientific Reports. She has received compensation as a Field Chief Editor for Frontiers in Molecular Biosciences and honoraria for participating in NIH peer review meetings.

## Author contributions

All listed authors made substantial contributions to the following: (1) the conception and design of this study, or the analysis and interpretation of data; (2) drafting the article or critically revising its important intellectual content; and (3) final approval of the version submitted. The contributions of individual authors were as follows. CG methodology, supervising, formal analysis, writing first original draft, preparing figures; writing‐review & editing; RK proposed, initiated, and helped design the study, utilized the Redox Biology Laboratory as a service contract to conduct the research, writing‐review & editing, and served as a study advisor. All authors have agreed on the final version of this study.

## Data Availability

The study did not generate new unique reagents. All data generated or analyzed during this study are included in this published article.
